# Global prevalence of osteoporosis among the world older adults: a comprehensive systematic review and meta-analysis

**DOI:** 10.1186/s13018-021-02821-8

**Published:** 2021-11-13

**Authors:** Nader Salari, Niloofar Darvishi, Yalda Bartina, Mojdeh Larti, Aliakbar Kiaei, Mahvan Hemmati, Shamarina Shohaimi, Masoud Mohammadi

**Affiliations:** 1 Department of Biostatistics, School of Health, Kermanshah University of Medical Sciences, Kermanshah, Iran; 2 Student research committee, Kermanshah University of Medical Sciences, Kermanshah, Iran; 3 Department of Translation Studies, Faculty of Literature, Istanbul University, Istanbul, Turkey; 4 Department of Computer Engineering, Sharif University of Technology, Tehran, Iran; 5 Department of Biology, Faculty of Science, University Putra Malaysia, Serdang, Selangor Malaysia; 6 Department of Nursing, School of Nursing and Midwifery, Kermanshah University of Medical Sciences, Kermanshah, Iran

**Keywords:** Prevalence, Osteoporosis, Elders, Systematic review, Meta-analysis

## Abstract

**Background:**

Osteoporosis is one of the most common bone system diseases that is associated with an increased risk of bone fractures and causes many complications for patients. With age, the prevalence of this disease increases so that it has become a serious problem among the elders. In this study, the prevalence of osteoporosis among elders around the world is examined to gain an understanding of its prevalence pattern.

**Methods:**

In this systematic review and meta-analysis, articles that have focused on prevalence of osteoporosis in the world’s elders were searched with these key words, such as Prevalence, Osteoporosis, Elders, Older adult in the Science Direct, Embase, Scopus, PubMed, Web of Science (WoS) databases and Google Scholar search engine, and extracted without time limit until March 2020 and transferred to information management software (EndNote). Then, duplicate studies were eliminated and the remaining studies were evaluated in terms of screening, competence and qualitative evaluation based on inclusion and exclusion criteria. Data analysis was performed with Comprehensive Meta-Analysis software (Version 2) and Begg and Mazumdar test was used to check the publication bias and I^2^ test was used to check the heterogeneity.

**Results:**

In a review of 40 studies (31 studies related to Asia, 5 studies related to Europe and 4 studies related to America) with a total sample size of 79,127 people, the prevalence of osteoporosis in the elders of the world; 21.7% (95% confidence interval: 18.8–25%) and the overall prevalence of osteoporosis in older men and women in the world, 35.3% (95% confidence interval: 27.9–43.4%), 12.5% (95% confidence interval: 9.3–16.7%) was reported. Also, the highest prevalence of osteoporosis in the elders was reported in Asia with; 24.3% (95% confidence interval: 20.9–28.1%).

**Conclusion:**

The results of the present study showed that the prevalence of osteoporosis in the elders and especially elders' women is very high. Osteoporosis was once thought to be an inseparable part of elders’ lives. Nowadays, Osteoporosis can be prevented due to significant scientific advances in its causes, diagnosis, and treatment. Regarding the growing number of elderly people in the world, it is necessary for health policy-makers to think of measures to prevent and treat osteoporosis among the elders.

## Background

Osteoporosis is characterized by a decrease in bone mass and destruction. According to an internationally agreed definition, people with BMD ≤  − 2.5 have a standard deviation less than the average healthy young population with osteoporosis [1]. In the last century, the average life expectancy of people has increased because of the increase in safety, life expectancy, and observance of health principles. As a result, the elderly population has expanded significantly [2]. According to the WHO, the elderly population will reach 12 billion by 2025 [3]. Aging is associated with chronic diseases, disabilities and cognitive decline [4]. Hypertension, sleep disorders, malnutrition, obesity, and osteoporosis and an increased risk of falls are other problems associated with aging [2, 5–8]. Therefore, the costs of treatment and social support are increasing day by day [4]. Osteoporosis is the most common metabolic disease, especially in the elders [9–11]. The prevalence of osteoporosis among the elders in 2020 in Spain and China was reported to be 39.3% and 39.4%, respectively [12, 13]. This amount was reported 49% in Nepal in 2019, 11% in Taiwan and 7.9% in Iran [9, 14, 15]. Female gender, age, marital status, history of peptic ulcer and fracture, and Osteoarthropathy are associated with osteoporosis in the elders [10].

Osteoporosis and osteoporotic fractures directly and indirectly impose a high cost on the global economy [16]. The annual cost of osteoporosis to the US health care system is at least $ 5–10 billion [17]. Osteoporosis increases the risk of fractures. Fractures can lead to decreased quality of life, hospitalization, disability and increased mortality [18, 19]. Osteoporotic fractures, especially vertebral fractures, can be associated with chronic debilitating pain. One in five patients with a pelvic fracture dies within a year [20]. Bone fractures make daily activities difficult. Only one-third of patients with fractures return to their previous level of function, and one-third of these patients require hospitalization in nursing homes [20].

In addition to fractures, osteoporosis can increase hospitalization rates due to associated secondary complications [21]. There are more than 8.9 million osteoporotic fractures worldwide. In other words, an osteoporotic fracture occurs every three seconds [22]. In the USA, about 1.5 million fractures occur due to osteoporosis each year [17]. It is estimated that one in three women and one in five men over the age of 50 suffer osteoporotic fractures [20]. More than one-third of adult women suffer from one or more osteoporotic fractures [23]. Due to the rapid increase in the average age of the population, an increase in the number of people with osteoporosis and, consequently, an increase in the number of fractures due to osteoporosis can be predicted [16]. The elderly population suffers more than others from the complications of osteoporosis [4]. The prevalence of osteoporosis among the elders was reported 36.1% in India. The figures were reported 1.6%, 19%, and 49% in Canada, Denmark, and Nepal, respectively [1, 14, 24, 25]. As can be seen, the prevalence of osteoporosis in the elders varies greatly in different countries and there is no accurate idea of the overall prevalence of osteoporosis in the world. Accordingly, the aim of this study was to investigate the prevalence of osteoporosis in the world’s elders through systematic review and meta-analysis.

## Methods

This study was conducted in accordance with the criteria of the Preferred Reporting Items for Systematic Reviews and Meta-Analyses (PRISMA) [26]. Based on which, systematic search of databases, organization of documents for review, selection of studies in accordance with the criteria defined by the authors, information extraction, analysis and finally the presentation of the final report were implemented.

### Search strategy

Systematic search of articles was performed in Google Scholar, Science Direct, Scopus, Web of science (WoS), PubMed, SID, Magiran databases. The keywords used for the search in this study were selected based on published preliminary studies and also Medical Subject Headings (MESH Terms) in the reviewed database. Also, a detailed study of the questions in this study and the keywords were selected according to PECO criteria [27].

PECO criteria included Participants: In this study, men and women older adults [28], Exposure: among all the elders, the elders with osteoporosis were examined, Comparison: Osteoporosis was considered in the elders of different communities, Outcomes: The overall prevalence of osteoporosis was reported by gender and continent. The selected keywords in this study were in English and their Persian equivalents were used in Persian databases. These keywords included Prevalence, Osteoporosis, Elders, Older adult. The Boolean search method was also used to combine the keywords. The search was conducted in various databases without time limit and until March 2020. References to past related studies and the Google Scholar search engine were also further explored to find relevant empirical studies.

### Inclusion and exclusion criteria

Inclusion criteria included cross-sectional studies that focused on the prevalence of osteoporosis in the elders, studies that have the full text available and the information in the present study, and exclusion criteria included observational studies such as control case and cohort studies, case report studies, case series, review studies, intervention and clinical trial studies.

### Selection of studies

After collecting the studies researched in EndNote software, the studies were started by the authors. Evaluations in this study were performed independently and blinded. In order to keep the information of the authors of the article anonymous, the journal title and the author name(s) were removed from the review list of articles, and then, the full text of the article was provided to reviewers. Initially, two researchers (ND and ML) reviewed the titles and abstracts of articles (according to inclusion criteria). In case of disagreement among the researchers regarding each of the articles, the third party (MM) reviewed and provided the final opinion regarding that study. Then, the full text of the studies confirmed in the initial evaluation was reviewed by the same researchers in terms of criteria defined according to the PECO criterion.

### Quality evaluation

The quality of confirmatory studies in the previous stages was measured by the methodological quality assessment tool of observational studies. The STROBE checklist [29] was used in this study. This checklist examines various aspects of writing a study, including title, problem statement, study objectives, study type, statistical population, sampling method, determining the appropriate sample size, defining variables and procedures, study data collection tools, statistical analysis methods and findings. Since the surveys in this checklist are done using 32 different fields. A score was assigned in the range of 0–32 to the studies. Due to the fact that in this systematic review, studies with good or average quality were included in the analysis, articles that received a score of 16 and above were selected by the authors, and studies with a score of less than 16 were considered to be of poor quality and excluded.

### Data extraction and analysis

Data were extracted through pre-designed forms. Various criteria such as demographic information (first author, year of publication, country, continent, study population, age mean) and the number of people with osteoporosis in general and by population were extracted and entered into the relevant forms, and Comprehensive Meta-Analysis software (Version 2) was used to analyze the data. Due to the high number of studies reviewed in this systematic review, the Begg and Mazumdar test at a significance level of 0.1 and the corresponding Funnel plot were used to investigate the publication bias. The *I*^2^ (%) test was used to assess the heterogeneity of the selected research works. Finally, using meta-regression test, the relationship between the prevalence of osteoporosis in the elders with the sample size, year of publication and age of participants in the study and analysis of meta-analysis by continent and sex was also investigated.

## Results

**Fig. 1:**
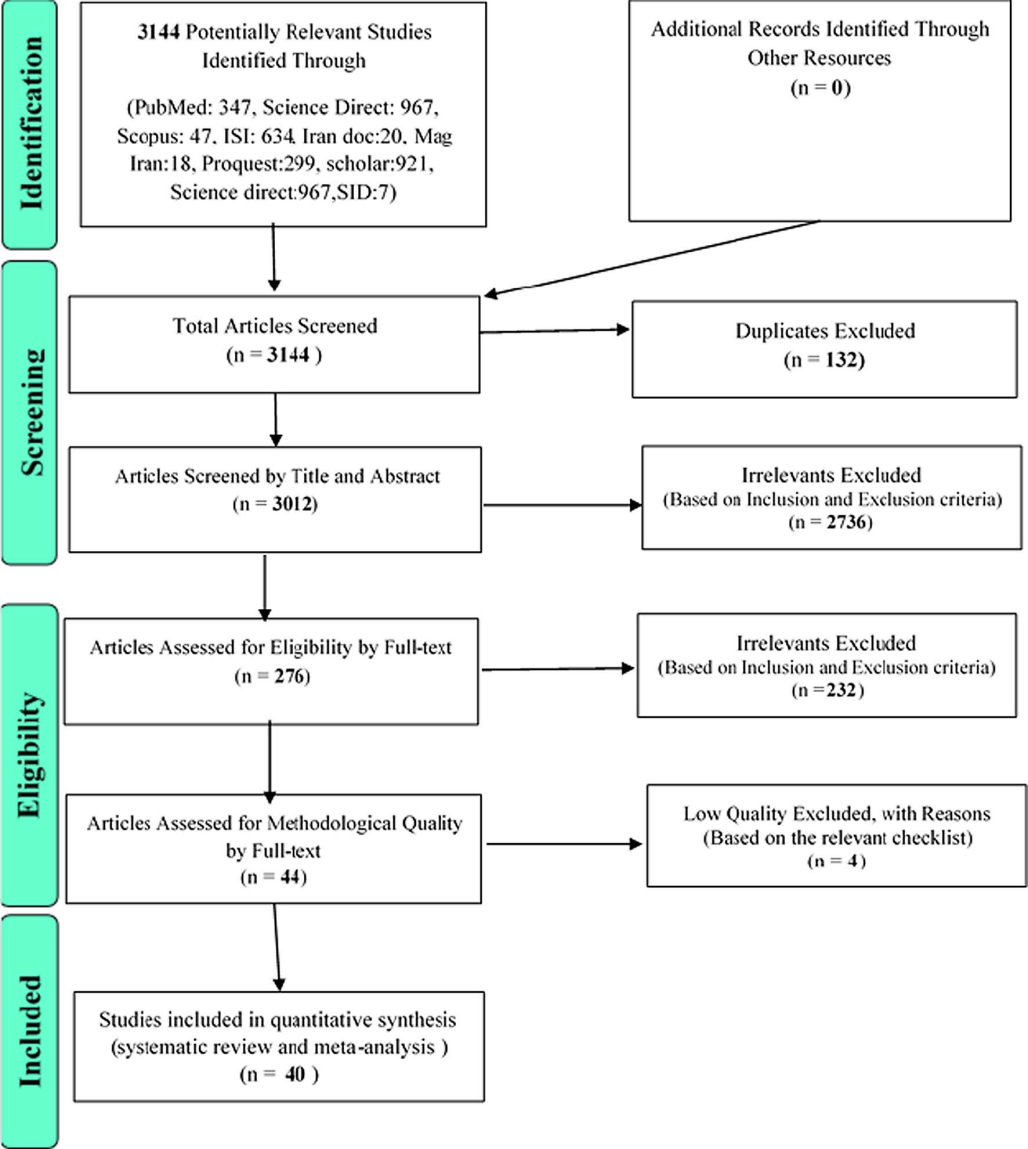
The flowchart on the stages of including the studies in the systematic review and meta-analysis (PRISMA 2009)

In this systematic review and meta-analysis, studies on the prevalence of osteoporosis in the world without time limit until March 2020 and according to PRISMA guidelines were systematically reviewed. Based on the initial search in the database, 3144 possible related articles were identified and transferred to the information management software (EndNote). Out of a total of 3144 identified studies, 132 were duplicate and were excluded. In the screening phase of the remaining 3012 studies, 2736 articles were excluded through the study of titles and abstracts based on inclusion and exclusion criteria. In the competency evaluation stage, out of the remaining 276 studies, 232 articles were excluded by reading the full text of the article based on inclusion and exclusion criteria due to irrelevance. In the qualitative evaluation stage, by studying the full text of the article and based on the score obtained from the STROBE checklist, out of the remaining 44 studies, 4 articles that had poor methodological quality were excluded. Finally, 40 studies entered the final analysis (Fig. 1).

The results of a systematic review of the studies in Tables 1 and 2 were reported based on the osteoporosis screening indices as well as the country in which the study was conducted. The lowest and highest sample sizes were related to the studies of Bakir et al. (2018) (*n* = 38) [30] and Lau et al. (2015) (*n* = 12,401) [31].

**Table 1:** Data on studies included in the meta-analysis

Number	First author	Year	Region	Age range	Total sample size	Total prevalence of osteoporosis
1	Ana Carolina Veiga Silva [32]	2015	Brazil	≥ 66	468	25.4
2	Antonio Juan [12]	2020	Spain	65	731	39.3
3	Anuk Kruavit [33]	2012	Thailand	75.2	93	47.3
4	B.R. Nielsen [1]	2020	Denmark	75	529	19
5	Bi Bi Fatemeh Nobakht Motlagh Ghochani [34]	2013	Iran	56.4	430	34.1
6	Carlos Mautalen [35]	2016	Argentina	≥ 50	5448	18.7
7	Chi‐Hua Ko [15]	2018	Taiwan	–	3144	11
8	Daisuke Asaoka [36]	2015	Japan	63.2	255	16.8
9	Dong-Hyeon Lee [37]	2013	Korea	55	1727	23.9
10	Edith Ming Chu Lau [31]	2015	China	50–89	12,401	22.5
11	Esad Alibasic [38]	2013	Bosnia And Herzegovina	70	711	8.8
12	Eun Jung Park [39]	2014	Korea	≥ 60	4538	33.8
13	Khurshid A. Bhat [40]	2018	India	68	241	19.9
14	Kok-Yong Chin [41]	2016	Malaysia	63.38	645	10
15	Kyae Hyung Kim [42]	2012	Korea	≥ 50	2870	39
16	Kyung-Shik Lee [43]	2014	Korea	≥ 60	6864	32.9
17	Limin Tian [44]	2017	China	≥ 60	5160	10.8
18	M Maddah [45]	2011	Iran	50–75	706	15.5
19	Marie-Therese Puth [46]	2018	Germany	≥ 65	4418	12.2
20	Mohamed Adel Bakir [30]	2018	Syria	62	38	13.1
21	Narendra Kumar Chaudhary [14]	2019	Nepal	≥ 60	102	49
22	Neeraj Kumar Agrawal [47]	2013	India	62.61 (≥ 50)	200	8.5
23	P. Modagan [25]	2018	India	≥ 60	304	36.1
24	Paolo Bucciarelli [48]	2010	Italy	65.1 (38–87)	446	13.2
25	Parvin Cheraghi [9]	2018	Iran	74.9	1779	7.9
26	Po-Han Chen [49]	2017	Taiwan	66.7	941	16.7
27	Qian Zhang [13]	2020	China	81.2	565	39.4
28	Qiang Zeng [11]	2019	China	≥ 65	8479	30.2
29	Renu Gupta [50]	2012	Kuwait	≥ 60	1010	28.9
30	Robert Ferrari [24]	2015	Canada	70.5	557	1.6
31	Rongtao Cui [51]	2016	China	> 65	1394	9
32	Sabrina E Noel [52]	2018	Puerto Rico	≥ 60	438	12.1
33	Sahana Shetty [53]	2014	India	58	252	19.8
34	Sarath Lekamwasam [54]	2009	Sri Lanka	≥ 60	337	11.5
35	Xiao-Guang Cheng [55]	2007	China	≥ 50	5083	31
36	Yin-Fan Chang [56]	2016	Taiwan	74	368	35.1
37	Yixuan Ma [57]	2018	China	66.9	1168	61.6
38	Yong Jun Choi [58]	2012	Korea	≥ 60	3140	32.4
39	Zahra Pourhashem [59]	2012	Iran	68.39	193	32.1
40	Zhifeng Sheng [60]	2011	China	62 (50–82)	954	39.4

**Table 2:** Data on studies included in meta-analysis by gender

Number	First author	Year	Females’ number	Males’ number	Total prevalence of osteoporosis	Prevalence of osteoporosis in women	Prevalence of osteoporosis in men
1	B.R. Nielsen [1]	2020	297	323	101	22.2	10.8
2	Daisuke asaoka [36]	2015	135	120	43	27.4	5
3	Dong-Hyeon Lee Bakir [37]	2013	813	914	414	37.5	11.9
4	Eun Jung Park [39]	2014	2442	2096	1538	52.9	11.8
5	Kok-Yong Chin [41]	2016	362	283	65	7.5	13.4
6	Kyung-Shik Lee [43]	2014	3528	3336	2263	45	20.3
7	Limin Tian [44]	2017	1955	3205	560	15.4	8.1
8	P. Modagan [25]	2018	152	152	110	50.7	21.7
9	Qiang Zeng [11]	2019	4597	3882	2569	45.9	11.8
10	Rongtao Cui [51]	2016	908	486	126	13	1.6
11	Sabrina E Noel [52]	2018	315	123	53	14	7.3
12	Y. Lim [61]	2017	2775	1760	2050	61.9	18.8
13	Yixuan Ma [57]	2017	652	516	720	69.2	52.1
14	Yong Jun Choi [58]	2012	1816	1324	1018	52.6	11.6
15	Zahra Pourhashem [59]	2012	88	105	62	55.7	12.4

**Fig. 2:**
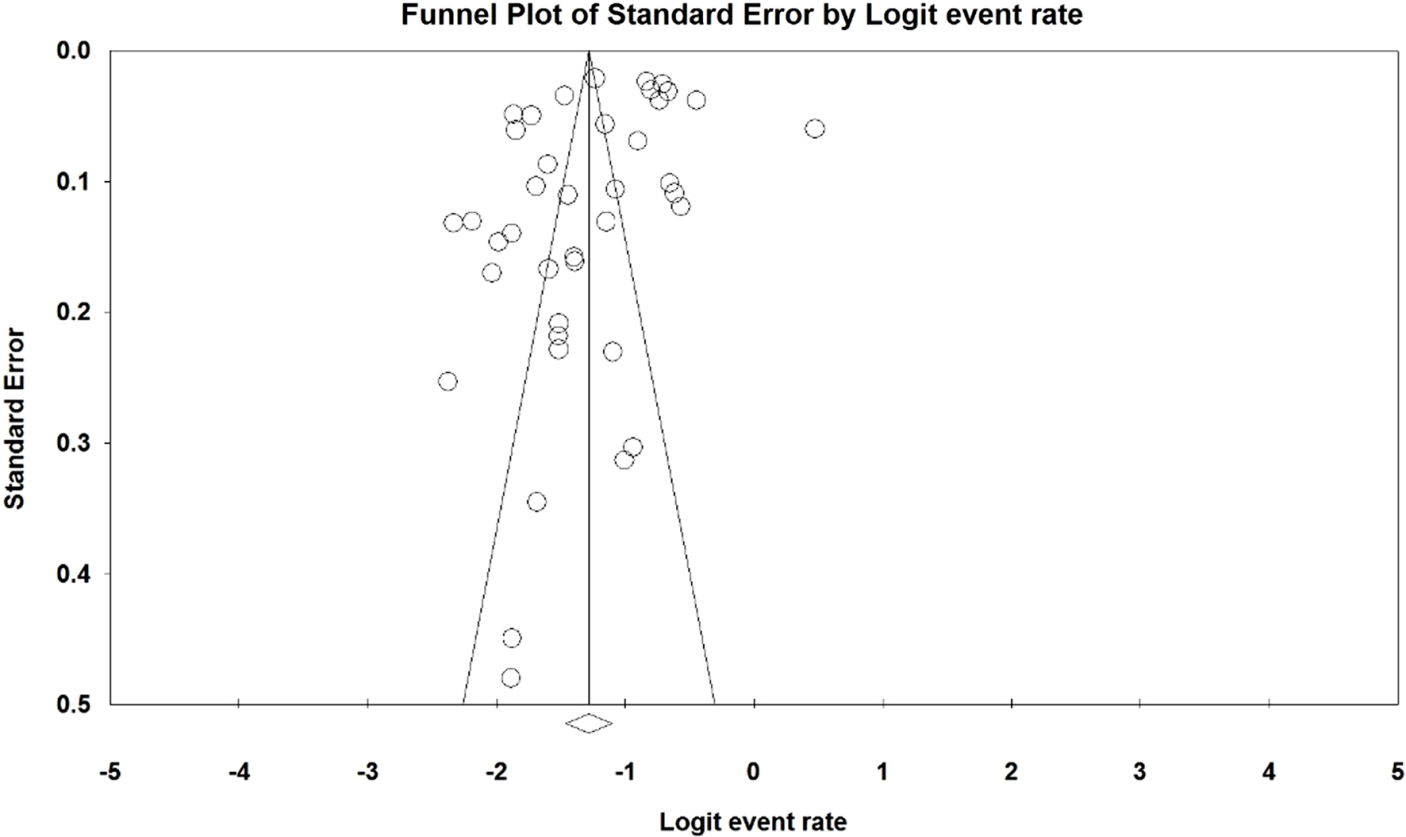
Funnel Plot Results of the prevalence of osteoporosis in the world’s elders

Based on the test results (*I*^2^: 99.07) and due to the heterogeneity of selected studies, a random effects model was used to combine the studies and share the prevalence estimate. The reason for heterogeneity between studies can be due to differences in sample size, sampling error, year of study or place of study. The probability of publication bias in results of the prevalence of osteoporosis in the elders in the world by funnel diagram and Begg and Mazumdar test at a significance level of 0.1 showed no publication bias of the prevalence in the present study (*P* = 0.278) (Fig. 2). Due to the high sample size studied in the study (79,127 elderly), in order to evaluate the results of publication bias, the Begg and Mazumdar test was used at a significance level of 0.1. However, the report of the results based on the Egger's test, which is more consistent with the funnel plot, was reported at the level of 0.05, which again, the publication bias was not significant (0.129).

**Fig. 3:**
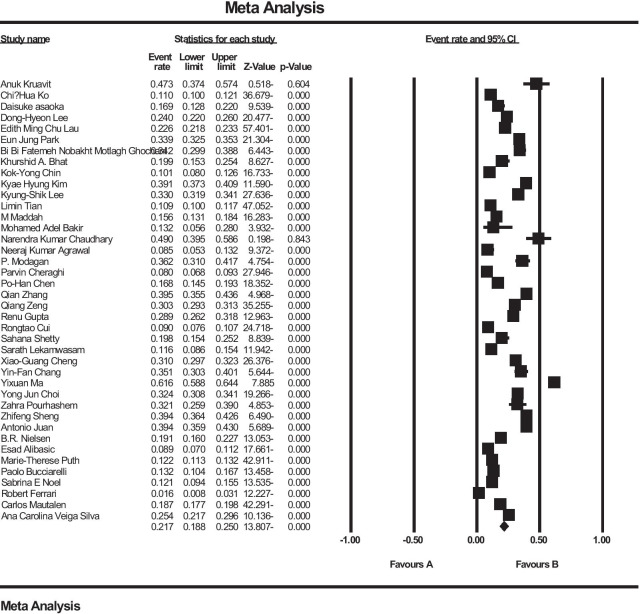
Prevalence of osteoporosis in the world’s elders and 95% confidence interval based on random effect model

In an investigation of 40 studies (31 studies in Asia, 5 studies in Europe, 4 studies in the Americas) with a total sample size of 79,127 people in the age range between 50 and 85 years, the prevalence of osteoporosis in the world elders; 21.7% (95% confidence interval: 18.8–25%) was obtained. The shape of Forrest Plot 3 reports the overall prevalence in the investigated studies, which shows the midpoint of each segment of the prevalence in each study, and the rhombus shape shows the prevalence in the population for the entire study (Fig. 3).

The results reported by the prevalence of osteoporosis in the elders by sex in Table 2 show that the highest and lowest prevalence of osteoporosis reported in elders women studied in China and in the 2017 Yixuan Ma study [57] with 69.2% (95% confidence interval: 65.5.8–72.6%) and in Malaysia and in the Kok-Yong Chin study [41], in 2016 with 7.5% (95% confidence interval: 5.2 –10.7%), and the highest and lowest prevalence of osteoporosis reported in elders men studied in China and in the Yixuan Ma study [57] in 2017 with 52.1% (95% confidence interval: 47.8–56.4%) and in Japan and in the Daisuke asaoka study [34], it was obtained in 2015 with 5% (95% confidence interval: 2.3–10.7%) (Table 2).

**Fig. 4:**
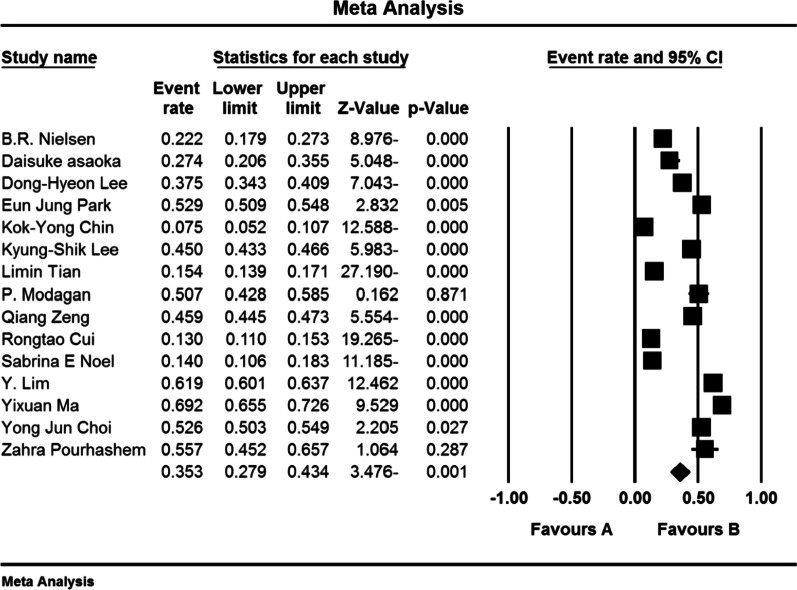
Prevalence of osteoporosis among elderly women worldwide and 95% confidence interval based on random effect model

The results of the meta-analysis reported in Fig. 4 report that the overall prevalence of osteoporosis in the elderly women of the world and the 95% confidence interval based on the random effects model was 35.3% (95% confidence interval: 27.9–43.4%). Heterogeneity of studies (*I*^2^: 99.2) and random effects model was used to evaluate the results. Also, the results of publication bias based on Begg and Mazumdar test at a significance level of 0.1 showed no publication bias in the present study (*P* = 0.276) (Fig. 4).

**Fig. 5:**
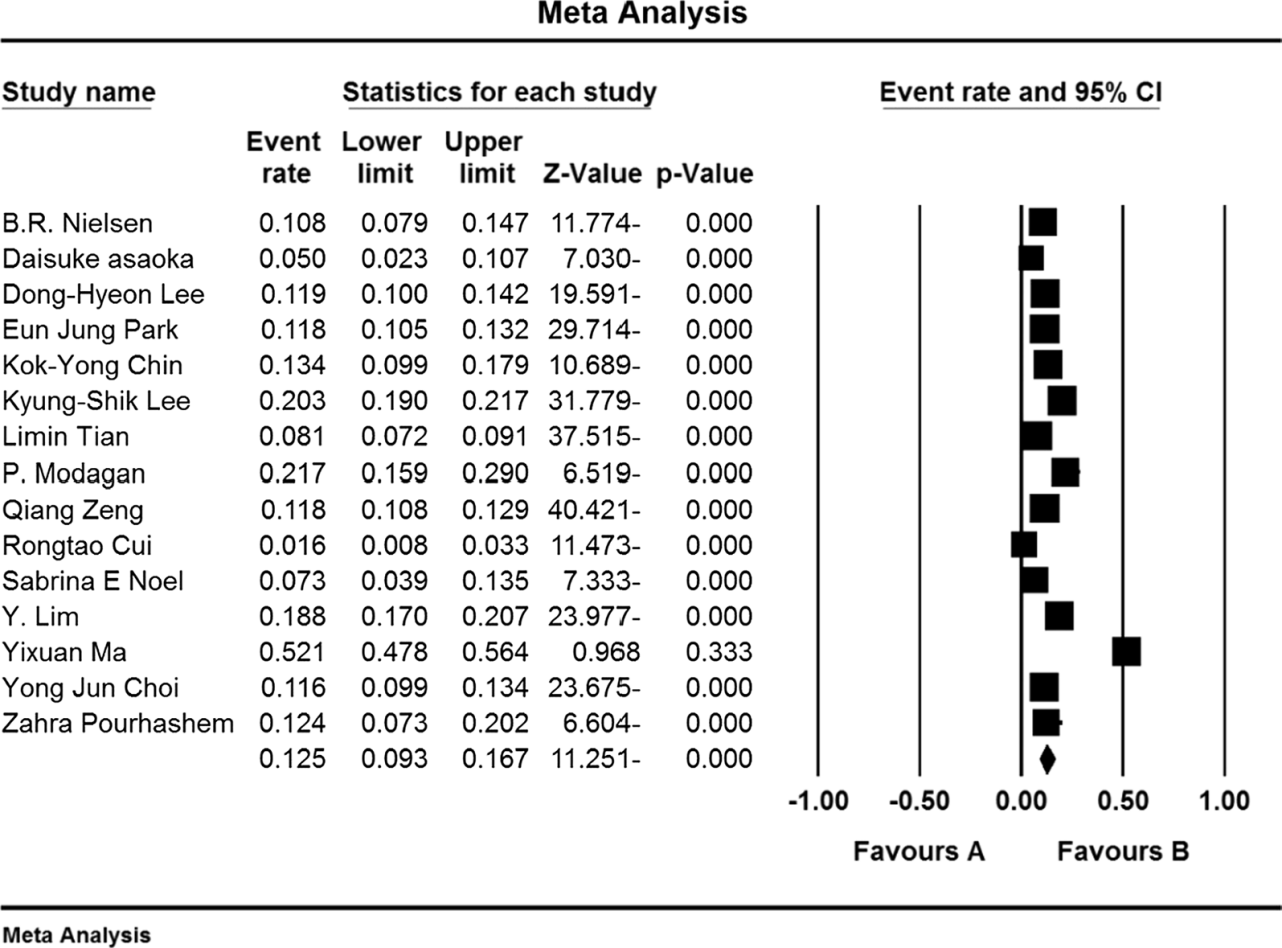
Prevalence of osteoporosis in the world’s elderly men and 95% confidence interval based on a random effect model

Also, the overall prevalence of osteoporosis in the elder’s men of the world and the 95% confidence interval based on the random effect model was reported to be 12.5% (95% confidence interval: 9.3–16.7%), heterogeneity of studies (*I*^2^: 98.1) and random effect model was used to evaluate the results. Also, the results of publication bias based on Begg and Mazumdar test at a significance level of 0.1 showed no publication bias in the present study (*P* = 1.000) (Fig. 5).

### Meta-regression test

**Fig. 6:**
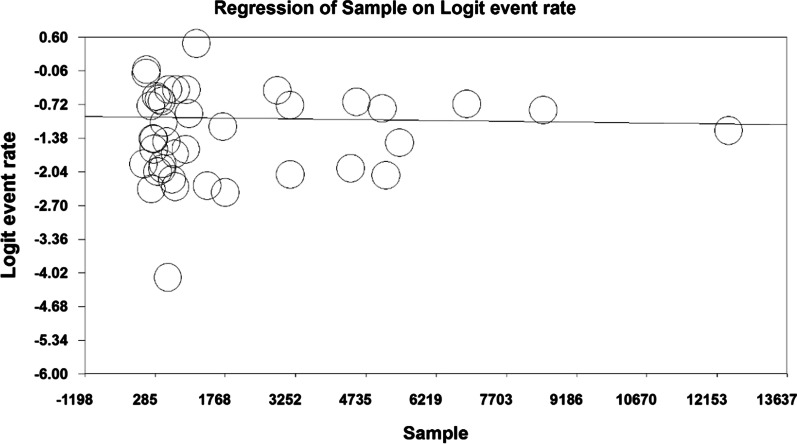
Meta-regression chart of the prevalence of osteoporosis in the elders of the world by sample size

**Fig. 7:**
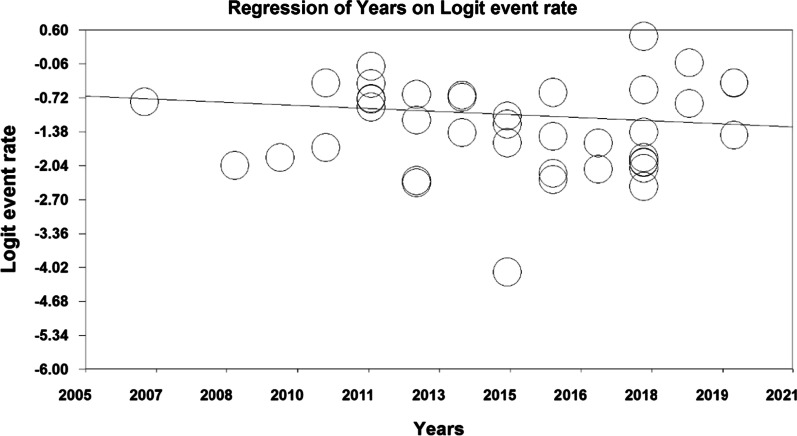
Meta-regression chart of the prevalence of osteoporosis in the elders of the world by year of study

**Fig. 8:**
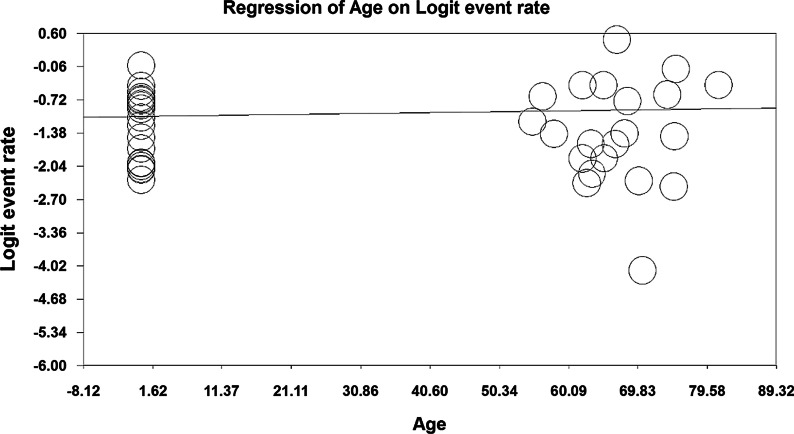
Meta-regression graph of the prevalence of osteoporosis in the elders of the world by age of study participants

In order to investigate the effects of potential factors in the heterogeneity of the prevalence of osteoporosis in the elders around the world, meta-regression was used on three factors: sample size, year of study and age of study participants (Figs. 6, 7, 8). According to Fig. 6, the prevalence of osteoporosis in the world elders decreases with increasing sample size, which is statistically significant (*P* < 0.05). This difference was also statistically significant (*P* < 0.05), but the results reported in Fig. 8 show that the prevalence of osteoporosis in the elders increases with age, which was also statistically significant (*P* < 0.05).

### Subgroup analysis

Table 3 reports the prevalence of osteoporosis in the elders of the world by different continents; these changes are reported in Asia, Europe, and the Americas, according to the results of this table, the highest prevalence of osteoporosis in the elders was Asia with 24.3% (95% confidence interval: 20.9–28.1%) (Table 3).

**Table 3:** Prevalence of osteoporosis in the elders of the world by different continents

Continents	Number of articles	Sample size	I^2^	Publication bias		Prevalence % (95% CI)
				Begg and Mazumdar Test	Egger’s test	
Asia	31	65,381	99.06	0.284	0.298	24.3 (95% CI: 20.9–28.1)
Europe	5	6835	99.8	1.000	0.831	16.7 (95% CI: 10.1–29)
America	4	6911	96.5	0.308	0.410	11.5 (95% CI: 5.4–19.1)

## Discussion

In a systematic review of 40 studies, the results of the meta-analysis of the present study report that the prevalence of osteoporosis in the world’s elders was obtained 21.7%. Based on the results of subgroup analysis, the prevalence of osteoporosis in Asia, Europe, and the USA was 24.3%, 16.7%, and 11.5%, respectively, with the highest prevalence in Asia.

Risk of osteoporosis in older people who need help, or have a functional defect is more than older people with favorable self-care activities [13]. In China in 2015, the prevalence of osteoporosis in people aged 50 and older was reported to be 16.96%, more than double the prevalence detected in 2006 [62].

In the Eastern Mediterranean, the prevalence of osteoporosis was reported 24.4%. The highest prevalence of osteoporosis was related to Saudi Arabia with 32.7% and the lowest prevalence was related to Kuwait with 15.1%. In Eastern Mediterranean countries, due to increased life expectancy, the prevalence of osteoporosis and its complications is increasing [63]. A case study by Zamani et al. [63], and Irani et al. [21] similarly reported issues such as low vitamin D and calcium intake, less sun exposure, and heavy smoking (more than 20 cigarettes per day), family history of osteoporosis; among the risk factors for osteoporosis in this area.

Based on the results of the present study, it was reported that the overall prevalence of osteoporosis in the elderly women of the world is 35.3% and in the elder’s men of the world is 12.5. Osteoporosis is more common in women than men. The results of many other studies were in line with the results of the study [16, 21, 62]. Female gender is an independent risk factor for osteoporosis in the elders. This increase may be related to a decrease in postmenopausal estrogen in women [13]. A history of fractures is the most important risk factor for osteoporosis in men [15]. Men generally have more bone mass and content than women. Also, men reach that high level of bone mass compared to women at older ages [64]. The prevalence of osteoporosis among women over 50 is estimated to be about 49% [65]. About one-tenth of women over the age of 60, one-fifth of women over the age of 70, two-fifths of women over the age of 80, and two-thirds of women over the age of 90 worldwide have osteoporosis [22]. Women over the age of 50 are 5 times more likely to develop osteoporosis than the normal population. In the study of Ma et al., the prevalence of osteoporosis was reported 69.1% in women and 52.1% in men [57]. In Nielsen et al. and Zeng et al., the prevalence of osteoporosis in men and women was reported to be 22.2%, 10.8%, 45.9% and 11.8%, respectively [1, 11].

There is some evidence that men are more likely than women to suffer from osteoporosis complications [16]. Women often get fractures 5–10 years earlier than men. Men are generally less likely to be screened for osteoporosis and less likely to seek treatment for fractures [66]. This difference is not due to diet, level of physical activity and weight, but may be related to differences in bone size between the sexes [66]. The prevalence of osteoporosis increases with age. People with osteoporosis were significantly older [13, 15, 21, 62, 63], had lower body weight, shorter height, and more previous fractures than people without osteoporosis [15]. The highest rate of bone loss occurs after the age of 65 [32]. In women, we will see menopause with age. Menopause activates rapid bone resorption in women. It is evident that this problem originates from ovarian insufficiency and can be prevented by estrogen replacement [67]. There is a balance between bone formation and resorption in men and women [32]. The rate of biochemical markers of bone resorption during menopause reaches 90%, while the biomarkers of bone formation reach 45%. This imbalance accelerates bone loss [67].

Based on a meta-analysis conducted worldwide and reviewing 86 studies with a sample size of 103,334,579 in the age range of 15 to 105 years, it was reported that the prevalence of osteoporosis in the world is 18.3% [68], which shows a high prevalence, while age is a high-risk factor for osteoporosis and Vitamin D insufficiency and reduced calcium absorption are common in the elderly [69, 70]. Osteoporosis is increasing due to increased life expectancy and an aging population [70]. The prevalence of fragility fractures increases with age, and hip fractures are more common in older age [70, 71]. In addition, it is very interesting to mention that 33.3% of women and 16.6% of men will sustain a hip fracture by their ninth decade, which is one of the most important causes of osteoporosis in old age.

Although, osteoporosis is more common in women, the risk of osteoporosis in men increases with age. Since most men do not experience overt hypogonadism with age, it was thought that the prevalence of osteoporosis in men was not related to sex hormone levels, but the results of several cross-sectional studies showed a positive and significant relationship between BMD and estrogen levels in men. Decreased serum estradiol levels are also associated with decreased bone density in men [67].

As mentioned, osteoporosis is characterized by a decrease in bone mass. Thus, the decrease in bone mass associated with aging increases the prevalence of osteoporosis in both men and women.

## Conclusion

The results of the present study showed that the prevalence of osteoporosis in the elders, and especially elders’ women, is very high. Osteoporosis was once thought to be an inseparable part of older people’s lives. Osteoporosis can be prevented today due to significant scientific advances in its causes, diagnosis and treatment. Regarding the growing number of elderly people in the world, it is necessary for health policy-makers to think of measures to prevent and treat osteoporosis among the elders.

## Data Availability

Datasets are available through the corresponding author upon reasonable request.

## Abbreviations

WHO: World Health Organization
WoS: Web of Science
STROBE: Strengthening the reporting of observational studies in epidemiology
PRISMA: Preferred Reporting Items for Systematic Reviews and Meta-Analysis
